# The Story of the Insane from Year to Year

**Published:** 1901-11-02

**Authors:** 


### THE STORY OF THE INSANE FROM YEAR TO YEAR.

(Thirteenth Series.)

### LONDON COUNTY ASYLUM AT HANWELL.

Here the numbers at the beginning of the year 1899 were 2,511, and at the end of December there was only a difference of 3. There were 430 first admissions, 95 readmissions, 209 recoveries, 47 discharged relieved, 36 discharged not improved, and 230 deaths. The average number resident during the year was 2,517. Excluding transfers and calculated on the year's admissions, the recoveries stand at the creditable percentage of 43·72, and calculated on the average number resident the deaths at 9·14 per cent. Of the year's deaths 119 were caused by cerebral and spinal diseases, general paralysis alone accounting for 48 of this number, consumption caused death in 27 cases, pneumonia in 6, bronchitis in 7, and enteritis in 10. In 23 of the admissions the insanity was caused by domestic trouble, in 32 by mental anxiety, in 10 by religion, in 83 by drink, and in 81 by hereditary influence, and Dr. Alexander points out that this latter cause is more potent among the women than among the men. As regards the deaths from colitis, it would seem that 30 patients were attacked; but there were in all about 80 cases of intestinal disturbance, the ulcerative colitis appearing chiefly among the senile and broken-down patients. The disease lost its hold when the cold weather set in, and Dr. Alexander says that "whatever the direct cause of ulcerative colitis may be proved to be, I think there is little doubt that the season of the year has much to do with favouring its development." We believe this to be true; but what is it; what is the nature of the infection, that is controlled by external temperature? and what other reasons can be assigned for the strangely erratic behaviour of this disease? At all events it would seem quite certain that imperfect drainage is not a direct cause, although instrumental in propagating the disease. The committee say, "it gives us much pleasure to again bear testimony to the efficient manner in which Dr. Alexander and the staff generally continue to carry out their responsible duties.' The weekly cost per head was 9s. 9d.

### THE NORWICH CITY ASYLUM.

Here there were 302 patients in residence on January 1st, 1899, and 307 on December 31st. There were 62 first admissions, 14 readmissions, 22 recoveries, 9 discharged relieved, 3 discharged not improved, and 37 deaths. The average number resident during the year was 300, and the total number of persons under treatment was 378. The recoveries stand at 30·98 per cent. of the admissions, and the deaths at 12·33 per cent. of the average number resident. Of the deaths 24 were caused by cerebral and spinal diseases, and 5 by consumption. Of the year's admissions 13 owed the development of insanity to various moral causes, 8 to intemperance in drink, 14 to hereditary influence, and 13 to "previous attacks." The latter always seems to us unsatisfactory as a stated cause, although it is found in all our lunacy statistics, and we think where practicable the cause of the first attack should also be given. Dr. Harris was able to obtain post-mortem examinations in 56 per cent. of the deaths. The consent of the relatives of the deceased is always obtained previous to these examinations being made in this asylum. In the omission of beer from the diet scale and in the substitution of other articles, a great improvement has been made, and it is one worthy of adoption in every public asylum in England. The committee "gladly congratulate Dr. Harris, Dr. Sykes, the heads of departments and those under them, upon the high efficiency of every department which has now been reached." The weekly cost per head is 8s. $11\frac{3}{4}$d. It is pointed out that this is nearly 1s. a week lower than other borough asylums, and that this result has not been obtained by lowering the diet or diminishing the comforts.

